# Effects of Red LED Irradiation in Enhancing the Mineralization of Human Dental Pulp Cells In Vitro

**DOI:** 10.3390/ijms24119767

**Published:** 2023-06-05

**Authors:** Ying Yang, Ok-Su Kim, Guo Liu, Bin-Na Lee, Danyang Liu, Wenqi Fu, Siyu Zhu, Jae-Seok Kang, Byunggook Kim, Okjoon Kim

**Affiliations:** 1 Dental Implant Center, School and Hospital of Stomatology, Wenzhou Medical University, Wenzhou 325027, China; dentistyang@wmu.edu.cn; 2 Department of Oral Pathology, School of Dentistry, Chonnam National University, Gwangju 61186, Republic of Korea; liudysunny@gamil.com (D.L.); fwq0369@gmail.com (W.F.); zhusiyu1994official@hotmail.com (S.Z.); jskang7041@gmail.com (J.-S.K.); 3 Department of Periodontology, School of Dentistry, Chonnam National University, Gwangju 61186, Republic of Korea; periodrk@jnu.ac.kr; 4 Hard-Tissue Biointerface Research Center, School of Dentistry, Chonnam National University, Gwangju 61186, Republic of Korea; 5 Department of Endodontics, School and Hospital of Stomatology, Wenzhou Medical University, Wenzhou 325027, China; dentistliu@wmu.edu.cn; 6 Department of Conservative Dentistry, School of Dentistry, Dental Science Research Institute, Chonnam National University, Gwangju 61186, Republic of Korea; bnlee13@jnu.ac.kr; 7 Department of Oral Medicine, School of Dentistry, Chonnam National University, Gwangju 61186, Republic of Korea; bkkimom@jnu.ac.kr

**Keywords:** red LEDI, photobiomodulation, dentin regeneration, ERK, P38

## Abstract

Dentin regeneration is the preferred method used to preserve dental pulp vitality after pulp exposure due to caries. Red light-emitting diode irradiation (LEDI), which is based on photobiomodulation (PBM), has been used to promote hard-tissue regeneration. However, the underlying mechanism still needs elucidation. This study aimed to explore the mechanism involved in red LEDI affecting dentin regeneration. Alizarin red S (ARS) staining revealed that red LEDI induced mineralization of human dental pulp cells (HDPCs) in vitro. We further distinguished the cell proliferation (0–6 d), differentiation (6–12 d), and mineralization (12–18 d) of HDPCs in vitro and treated cells either with or without red LEDI in each stage. The results showed that red LEDI treatment in the mineralization stage, but not the proliferation or differentiation stages, increased mineralized nodule formation around HDPCs. Western blot also indicated that red LEDI treatment in the mineralization stage, but not the proliferation or differentiation stages, upregulated the expression of dentin matrix marker proteins (dentin sialophosphoprotein, DSPP; dentin matrix protein 1, DMP1; osteopontin, OPN) and an intracellular secretory vesicle marker protein (lysosomal-associated membrane protein 1, LAMP1). Therefore, the red LEDI might enhance the matrix vesicle secretion of HDPCs. On the molecular level, red LEDI enhanced mineralization by activating the mitogen-activated protein kinase (MAPK) signaling pathways (ERK and P38). ERK and P38 inhibition reduced mineralized nodule formation and the expression of relevant marker proteins. In summary, red LEDI enhanced the mineralization of HDPCs by functioning to produce a positive effect in the mineralization stage in vitro.

## 1. Introduction

Dental pulp exposure is one of the consequences of progressive caries, the most common oral disease, which results in pulp infection and tooth loss. Previous studies have shown that dental pulp is a highly vascularized tissue and can form reparative dentin with some therapeutic intervention [1,2]. Therefore, inducing dentin regeneration to reseal pulp perforations is preferred to preserve pulp vitality and prolong tooth longevity. 

So far, dentin regeneration has mainly been induced using chemical compounds in clinical settings [3,4]. These compounds cover the exposed dental pulp and stimulate human dental pulp cells (HDPCs) to generate reparative dentin. However, chemical compounds may also be associated with cytotoxicity, tooth discoloration, and high cost [1]. Hence, therapeutic approaches with weak cytotoxicity, no complications, and low cost are necessary. 

Photobiomodulation (PBM), also called low-level light therapy (LLLT), is a noninvasive therapeutic approach. PBM can regulate cellular physiological and pathological reactions using a laser or a light-emitting diode (LED) [5]. Although some researchers insist that a laser is essential for PBM, increasing literature supports the opinion that PBM is not dependent on a laser or an LED [6]. Thus, LED irradiation (LEDI) is widely used in PBM due to its advantages of no laser safety consideration, easy use, and cost-effectiveness [7]. 

Previous studies have explored the positive effects of red LEDI. Besides its anti-inflammatory, anti-oxidative, skin-rejuvenation, and wound-healing properties, red LED has attracted attention for its tissue regeneration potential [7,8,9,10]. Cellular research reveals that red LEDI can induce osteogenic/odontogenic differentiation and mineralization of stem cells in vitro [11,12]. Moreover, clinical studies and animal experiments demonstrate that red LEDI can accelerate bone healing in vivo [13,14]. 

However, the mechanism of red LEDI in hard-tissue regeneration is still unclear. Current evidence indicates that hard-tissue regeneration depends on stem or stem-like cell dynamics involving cell proliferation, differentiation, and mineralization. Some authors suggest that red LEDI can modulate stem-cell proliferation, whereas others claim that red LEDI does not alter cell proliferation but enhances cell differentiation and mineralization [15,16,17,18]. Moreover, the timing of red LEDI applications is also controversial. Several studies show applications of red LEDI only once when starting cell culture, while other research reveals multiple red LEDI applications [12,17,19].

In this study, we firstly identified that red LEDI promoted the mineralization of HDPCs. Further, we distinguished the cell proliferation, differentiation, and mineralization stages of HDPCs in vitro and found that red LEDI primarily enhanced the mineralization of HDPCs in the matrix mineralization phase. 

## 2. Results

### 2.1. Effect of Red LEDI on Cell Viability of the HDPCs

**Figure 1:**
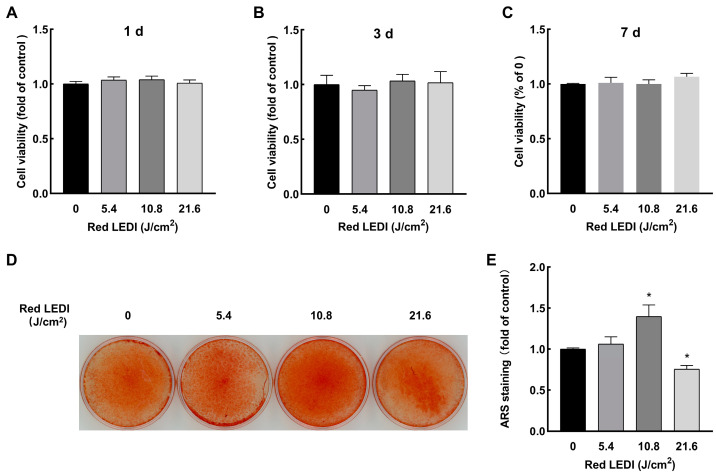
Effect of red LEDI on cytotoxicity and mineralization of HDPCs. (**A**–**C**) Different energy densities of red LEDI did not show cytotoxicity on the HDPCs after 1, 3, and 7 d culturing. (**D**) An amount of 10.8 J/cm^2^ red LEDI increased the mineralization of HDPCs. (**E**) Quantitative analysis of ARS staining. Bars show means *±* standard deviation (* *p* < 0.05, compared to 0 J/cm^2^. LEDI: light-emitting diode irradiation; HDPCs: human dental pulp cells; ARS: alizarin red S).

A cell viability assay was performed to assess the cytotoxicity of red LEDI on HDPCs. Based on our previous study [20], cells were treated with 0, 5.4, 10.8, and 21.6 J/cm^2^ red LEDI. After 1, 3, or 7 d culturing, the irradiation groups did not show cytotoxicity (Figure 1A–C).

### 2.2. Effect of Red LEDI on Mineralization of the HDPCs

ARS staining was performed to examine the effect of red LEDI on the calcification of HDPCs. After 21 d culturing with red LEDI every 3 days, calcification of the HDPCs in the 5.4 J/cm^2^ group was not affected, and the 21.6 J/cm^2^ group showed decreased mineralization of HDPCs. However, 10.8 J/cm^2^ red LEDI increased the mineralization of HDPCs. (Figure 1D,E). Therefore, 10.8 J/cm^2^ red LEDI was chosen for further experiments.

### 2.3. Identification of Cell Proliferation, Differentiation, and Mineralization Stages of the HDPCs In Vitro

**Figure 2:**
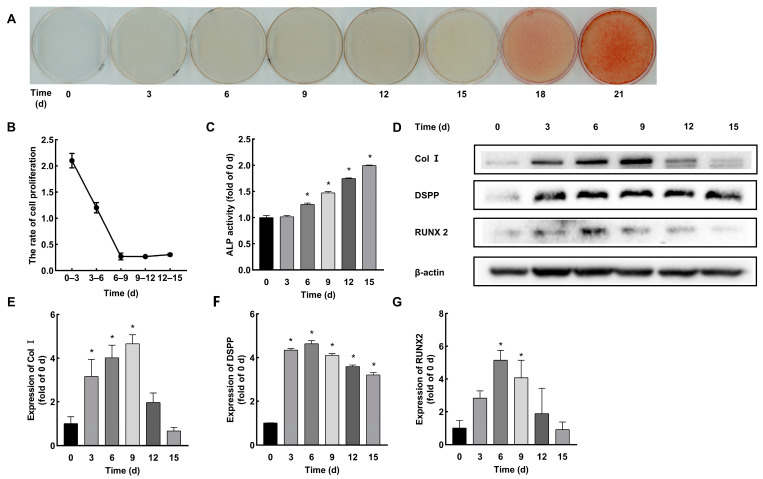
Identification of cell proliferation, differentiation, and mineralization stages of the HDPCs in vitro. (**A**) ARS staining every 3 days in vitro. Mineralized nodule formation was observed from 15 d and progressively increased over time. (**B**) The proliferation rate of the HDPCs. Cells exhibited rapid growth in the first 6 days but steadily grew from 6 d to 15 d. (**C**) ALP activity assay every 3 days. ALP activity increased significantly after 6 d and then enhanced gradually over time. (**D**) The expression level of Col I, DSPP, and RUNX2. (**E**–**G**) Quantitative analysis of ALP, Col I, DSPP, and RUNX2 expression. (Results were normalized to 0 d. * *p* < 0.05 compared to 0 d. ALP: alkaline phosphatase; Col I: collagen I; DSPP: dentin sialophosphoprotein; RUNX2: runt-related transcription factor 2.)

To identify the cell proliferation, differentiation, and mineralization stages of the HDPCs in vitro, we first performed ARS staining every 3 days during the 21 days of cell culturing. Mineralized nodule formation was observed from 15 d and progressively increased over time (Figure 2A). Next, the rate of cell proliferation was measured (Figure 2B). After rapid cell proliferation in the first 6 days, the HDPCs grew steadily from 6 d to 15 d. The study examined the expression level of differentiation markers (ALP, Col I, DSPP, RUNX2). The results showed significantly increased ALP activity after 6 d that gradually enhanced over time (Figure 2C). Western blot revealed upregulated expression of Col I and RUNX2 at 3 d that regulated to low after 12 d (Figure 2D–G). DSPP increased at 3 d but showed high expression after 12 d (Figure 2D,F). Based on our results above, we distinguished the cell proliferation, differentiation, and mineralization stages of the HDPCs as 0–6 d, 6–12 d, and 12–18 d, respectively.

### 2.4. Red LEDI in the Mineralization Stage Enhanced the Calcification of the HDPCs

To further investigate when the red LEDI mainly affects the mineralization of HDPCs, cells were treated either with or without red LEDI in the cell proliferation, differentiation, and mineralization stages. The experiment was divided into five groups: negative control group (Nega, without red LEDI), positive control group (Posi, with red LEDI treatment in the proliferation, differentiation, and mineralization stages), proliferation group (Pro, with red LEDI treatment only in the proliferation stage), differentiation group (Diff, with red LEDI treatment only in the differentiation stage), and mineralization group (Mine, with red LEDI treatment only in the mineralization stage). 

**Figure 3:**
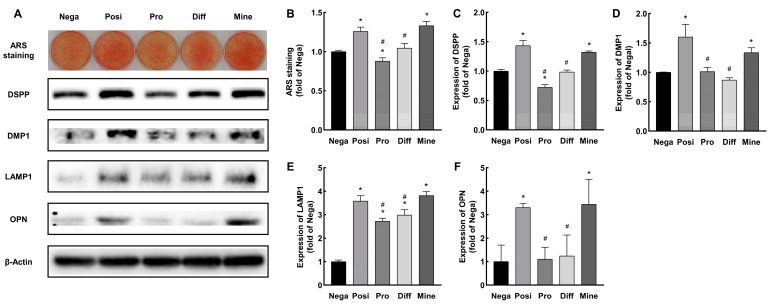
Red LEDI in the mineralization stage enhanced the calcification of HDPCs. (**A**) Compared to the Nega group, mineralized deposition increased in the Posi and Mine groups but decreased in the Pro group. The Posi and Mine groups showed upregulation in the expression of DSPP, DMP1, OPN, and LAMP1. The Pro group showed increased expression of LAMP1 and decreased expression of DSPP, but DMP1 and OPN were unaffected. The Diff group showed enhanced LAMP1 expression but no change in DSPP, DMP1, and OPN expression. Compared to the Posi group, the Mine group showed similar mineralized nodule formation and odontogenic relative proteins expression (DSPP DMP1, LAMP1, and OPN), which were significantly reduced in the Pro and Diff groups. (**B**–**F**) Quantitative analysis of ARS staining and DSPP, DMP1, LAMP1, and OPN expression. (Results were normalized to the Nega group. * *p* < 0.05 compared to the Nega group, # *p* < 0.05 compared to the Posi group; DMP1: dentin matrix protein 1; LAMP1: lysosomal-associated membrane protein 1; OPN: osteopontin.)

ARS staining revealed that mineralized nodule formation increased in the Posi and Mine groups but decreased in the Pro group compared to the Nega group. Compared to the Posi group, the Mine group showed similar mineralized nodule formation, which was significantly reduced in the Pro and Diff groups (Figure 3A,B).

We further examined the expression levels of three dentin matrix marker proteins (DSPP, DMP1, and OPN) and an intracellular secretory vesicle marker protein (LAMP1) in each group (Figure 3A,C–F). Compared to the Nega group, the Posi and Mine groups upregulated the expression of DSPP, DMP1, OPN, and LAMP1. The Pro group showed increased expression of LAMP1 and decreased expression of DSPP, but DMP1 and OPN were unaffected. The Diff group showed enhanced LAMP1 expression but no change in DSPP, DMP1, and OPN expression. Compared to the Posi group, the DSPP, DMP1, LAMP1, and OPN expression of the Pro and Diff groups was reduced, but these proteins in the Mine group were still highly expressed. 

Our findings showed that red LEDI treatment in the mineralization stage promoted the mineralization of HDPCs.

### 2.5. Red LEDI in the Mineralization Stage Enhanced the Calcification of the HDPCs by Activating ERK and P38 Signaling Pathways

**Figure 4:**
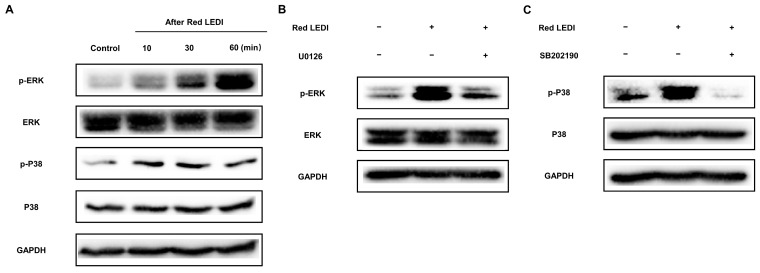
ERK and P38 were activated by red LEDI but inhibited by U0126 and SB202190, respectively. (**A**) The expression of p-ERK and p-P38 was increased after red LEDI treatment within 60 min. (**B**) p-ERK was inhibited by ERK inhibitor, U0126. (**C**) p-P38 was inhibited by P38 inhibitor, SB202190.

To elucidate the molecular mechanism of red LEDI inducing the mineralization of HDPCs, we explored the role of the mitogen-activated protein kinase (MAPK) signaling pathways (ERK and P38). During the mineralization stage, p-ERK and p-P38 were activated after red LEDI application within 60 min (Figure 4A). Moreover, after pretreatment with the inhibitors of ERK (U0126, Cell Signaling Technology, USA) and P38 (SB202190, USA), the activation of ERK and P38 was inhibited significantly (Figure 4B,C). 

**Figure 5:**
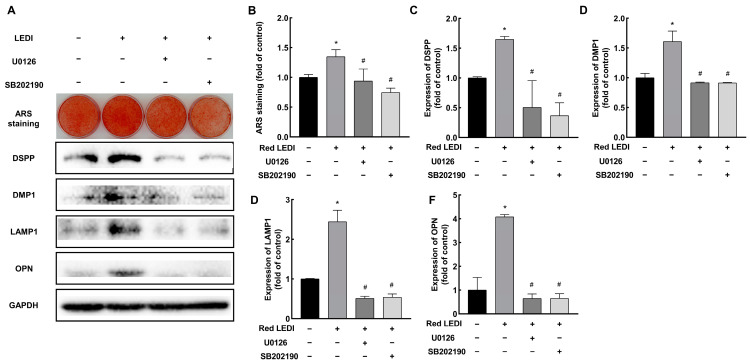
Red LEDI enhanced the mineralization of the HDPCs by activating ERK and P38 signaling pathways. (**A**) After the pretreatment with U0126 and SB202190, mineralized deposits and proteins (DSPP, DMP1, LAMP1, and OPN) decreased compared to the red LEDI group. (**B**–**F**) Quantitative analysis of ARS staining and of DSPP, DMP1, LAMP1, and OPN expression. (Results were normalized to the control group. * *p* < 0.05 compared to the control group, # *p* < 0.05 compared to the red LEDI group.)

We then examined whether red LEDI induced the calcification of HDPCs, by activating ERK and P38. Pretreatment with U0126 or SB202190 reduced the formation of mineralized nodules compared to the red LEDI group (Figure 5A,B). The mineralized associated proteins also demonstrated similar results. Compared to the red LEDI group, the expression of DSPP, DMP1, LAMP1, and OPN in the ERK and P38 groups showed significant downregulation. 

## 3. Discussion

The present study evaluated the effect of red LEDI on the proliferation, differentiation, and mineralization stages of HDPCs in vitro and found that red LEDI primarily enhanced the mineralization of HDPCs in the matrix mineralization phase by activating the ERK and P38 MAPK signaling pathways.

Previous studies have suggested that red light irradiation induces mineralization because it promotes the proliferation and differentiation of stem or stem-like cells [19,21,22]. Interestingly, our data indicated that red LEDI enhanced the mineralization of HDPCs by functioning in the mineralization stage. ARS staining revealed enhanced mineral deposition in the Mine group; however, mineral deposition was unaffected in the Diff group and was slightly reduced in the Pro group. PBM plays a different role at various radiant exposures (dose-dependent response). After reviewing the literature, we found that red light promoted proliferation at an energy density of 1–4 J/cm^2^ and osteogenic/odontogenic differentiation at 2–6 J/cm^2^ [10]. However, 10.8 J/cm^2^ red LEDI was applied in our study. The high energy density of red LEDI might not promote the proliferation and differentiation of HDPCs but is beneficial for mineralization. Our cell-viability result verified that 10.8 J/cm^2^ red LEDI did not increase the proliferation of HDPCs. Furthermore, the low irradiance of our device (3 mW/cm^2^), mimicking sunlight, might be another reason. However, further exploration is necessary. 

We next examined the expression of the proteins DSPP, DMP1, OPN, and LAMP1. All these proteins were upregulated in the Mine group. DSPP, DMP1, and OPN belong to the small integrin-binding ligand N-linked glycoprotein family and play an important role during dentin mineralization [23,24]. DSPP is the precursor of dentin sialoprotein (DSP) and dentin phosphoprotein (DPP). Reports show that DSP functions as an initiator of dentin mineralization, but DPP promotes the maturation of the pre-dentin matrix [25]. Other studies have revealed that DMP1 and OPN are present during tooth development and orchestrate mineralized matrix formation [26,27]. LAMP1 is a marker of intracellular secretory vesicles and is involved in matrix vesicles (MVs) secretion. Previous studies have discussed the function of MVs on mineralization [28]. MVs transport the intracellular mineralization-promoting enzyme to the extracellular matrix and increase the inorganic phosphate level to initiate the nucleation of minerals. Therefore, we proposed that red LEDI enhanced the mineralization of HDPCs by upregulating the mineralization-associated protein and increasing the secretory ability of MVs.

Moreover, p-ERK and p-P38 were increased after red LEDI application, implying that red LEDI activated the ERK and P38 signaling pathways. Reports show that ERK and P38 play important roles during hard-tissue mineralization [29,30,31]. We assumed that ERK and P38 might be vital signaling pathways for red LEDI to regulate the mineralization of HDPCs. To verify this speculation, we pretreated with U0126 and SB202190, inhibitors of ERK and P38 mitogen-activated protein kinase, respectively. Our results showed that pretreatment with U0126 and SB202190 inhibited the activation of ERK and P38, consistent with other investigations [32,33,34]. Moreover, mineralized deposition and expression of proteins (DSPP, DMP1, OPN, and LAMP1) showed significant reduction. These data indicate that ERK and P38 are key signaling pathways for red LEDI to regulate the mineralization of HDPCs.

Dentin regeneration is a complex biological process involving intracellular and extracellular regulation. This study only investigated the effect of red LEDI on HDPCs but did not elucidate extracellular matrix regeneration. Thus, further studies are needed to explore the effect of red LEDI on the extracellular matrix. 

## 4. Materials and Methods

### 4.1. Cell Isolation and Cell Culture

Ethical approval was obtained from the ethical committee of Chonnam National University Dental Hospital (approval number: CNUDH-2022-006, 30 May 2022). Human dental pulp cells (HDPCs) were isolated from permanent teeth with patient approval following the protocol below. After extraction, the tooth was immersed in 4 °C Dulbecco’s phosphate-buffered saline (DPBS, WELGENE, Gyeongsangbukdo, Republic of Korea) containing 1% penicillin/streptomycin (WELGENE, Gyeongsangbukdo, Korea) and then transported to the laboratory for cell isolation. Dental pulp tissue was minced into 1 mm^3^ pieces and cultured with minimum essential medium-α (α-MEM, Gibco Invitrogen, Gaithersburg, MD, USA) containing 20% fetal bovine serum (FBS, Gibco Invitrogen, Gaithersburg, USA), 1% penicillin/streptomycin at 37 °C in a humidified atmosphere with 5% CO_2_. HDPCs between passages 3 and 6 were used in further experiments. 

The HDPCs were seeded at a density of 5 × 10^3^ cells/cm^2^ and cultured with odontogenic differentiation medium (OM, α-MEM containing 10% FBS, 1% penicillin/streptomycin, 50 μg/mL ascorbic acid (Sigma-Aldrich, St. Louis, MO, USA) and 10 mmol/L β-glycerophosphate (Sigma-Aldrich, St. Louis, MO, USA)). The medium was changed every 3 days.

### 4.2. Light Source and Irradiation

This study used a continuous-wave LEDI system (U-JIN LED, Gwangju, Republic of Korea) containing a cell incubator at a wavelength of 625 nm and irradiance of 3 mW/cm^2^. The distance between the light source and the sample surface was about 25 mm. The 625 nm LEDI was applied every 3 days.

### 4.3. Cell Viability Assay

A cell viability assay was performed using the EZ-Cytox Enhance cell viability assay kit (DoGenBio, Seoul, Republic of Korea). The HDPCs were seeded in 96-well plates and incubated for 24 h. Cells were irradiated for 0, 30, 60, and 120 min with total radiant exposure 0, 5.4, 10.8, 21.6 J/cm^2^, and cell viability was examined following the protocol below. Briefly, 100 µL EZ-Cytox solution was added to each well and incubated in a 5% CO_2_ incubator for 1.5 h at 37 °C. Optical density (OD) values of samples were measured at 450 nm using a microplate reader (BioTek, El Segundo, CA, USA).

### 4.4. Alizarin Red S (ARS) Staining

The HDPCs were seeded in a 35 mm petri dish and cultured with OM. Cells were rinsed with DPBS twice and then fixed with 4% paraformaldehyde for 15 min at room temperature. After washing with deionized H_2_O (diH_2_O) thrice, cells were stained using 2% ARS solution (Sciencell, Carlsbad, CA, USA) for 20–30 min at room temperature. The images were captured using a scanner (Epson, Yamagata, Japan). Samples were dissolved in 10% cetylpyridinium chloride (CPC, PH = 7.0) to quantify mineralized nodule formation. OD values were measured at 540 nm using a microplate reader.

### 4.5. Alkaline Phosphatase (ALP) Activity Assay

The HDPCs were seeded in a 60 mm petri dish and cultured with OM. Cells were lysed using radioimmunoprecipitation assay buffer (RIPA, Biosesang, Yongin, Republic of Korea) containing a protease inhibitor cocktail and phenylmethanesulfonyl fluoride (PMSF). The concentration of the whole-cell lysate was detected using a BCA protein assay kit (Thermo Fisher Scientific, Waltham, MA, USA). Equivalent samples were used to detect ALP activity using a pNPP ALP assay kit (ANASPEC, Fremont, CA, USA) following the manufacturer’s protocol. OD value was measured at 405 nm using a microplate reader.

### 4.6. Western Blotting

The whole-cell lysate was obtained according to the protocol mentioned above. The lysates were separated by sodium dodecyl sulfate-polyacrylamide gel electrophoresis (SDS-PAGE) and transferred to a polyvinylidene difluoride membrane (Millipore, Boston, MA, USA). After blocking with 5% nonfat milk dissolved in Tris-buffered saline with Tween 20 (TBS-T), the membranes were incubated with primary antibodies. The primary antibody for collagen I (Col I) was purchased from Abcam. The antibody for osteopontin (OPN) was purchased from Novus. The antibodies for dentin sialophosphoprotein (DSPP), dentin matrix protein 1 (DMP1), and runt-related transcription factor 2 (RUNX2) were purchased from Thermo Fisher Scientific. The antibodies for lysosomal-associated membrane protein 1 (LAMP1), β-Actin, ERK, phospho-ERK (p-ERK), P38, phospho-P38 (p-P38) were purchased from Cell Signaling Technology. The antibody for glyceraldehyde-phosphate dehydrogenase (GAPDH) was purchased from Santa Cruz Biotechnology. The secondary antibodies were anti-mouse IgG, anti-rabbit IgG, and anti-goat IgG (Thermo Fisher Scientific, IL, USA).

### 4.7. Statistical Analysis

The results from the independent experiments were expressed as mean ± SD. Statistical analysis of the experimental data was performed using the one-way ANOVA method. Data were considered statistically significant when the *p*-value was <0.05.

## 5. Conclusions

The present study demonstrates that red LEDI enhances the mineralization of HDPCs by affecting the mineralization stage in vitro, which provides a novel insight into the mechanism of dentin regeneration by red LEDI. 

## Data Availability

Data available on request due to restrictions, e.g., privacy or ethical.
